# *Berberis vulgaris* and its role in atherosclerosis improvement: A review of *in vitro* and *in vivo* data

**DOI:** 10.22038/ajp.2025.25922

**Published:** 2025

**Authors:** Mohammad Amin Momeni-Moghaddam, Morteza Rostamian, Reza Mohebbati

**Affiliations:** 1 *Department of Nutrition and Biochemistry, Faculty of Medicine, Social Determinants of Health Research Center, Gonabad University of Medical Science, Gonabad, Iran*; 2 *English Department, Faculty of Medicine, Gonabad University of Medical Science, Gonabad, Iran*; 3 *Nursing Research Center, Gonabad University of Medical Sciences, Gonabad, Iran*

**Keywords:** Berberis vulgaris, Cardiovascular, Atherosclerosis, Inflammation, Oxidative stress

## Abstract

**Objective:**

Atherosclerosis is a multifactorial condition influenced by various factors including inflammation and oxidative stress. Using drugs that reduce inflammation and oxidative stress is beneficial in preventing the formation and progression of atherosclerotic plaques. In light of the adverse effects associated with pharmaceutical interventions, natural compounds present a potentially safer therapeutic alternative for managing inflammation and oxidative stress. This study investigates the anti-inflammatory and antioxidant effects of *Berberis vulgaris* (*B. vulgaris*), not berberine, in relation to atherosclerosis.

**Material and methods:**

Databases such as PubMed, Web of Science, and Scopus were considered. The search terms were "*Berberis vulgaris*", "Cardiovascular", "Atherosclerosis", "Inflammation", "Oxidative stress", "Clinic", "Animal", "In vitro", "Cell line" and "Ingredient". The articles were reviewed from 2004 to 2024.

**Results:**

*B. vulgaris* known for its anti-inflammatory, antioxidant and immunomodulatory properties since it contains 22 alkaloid compounds, with berberine being the most prominent. Although berberine has been studied extensively in relation to atherosclerosis, its therapeutic use has been limited due to its poor oral bioavailability, which is less than 1%. Moreover, based on the literature the whole extract of B.* vulgaris* can be useful for atherosclerosis prevention.

**Conclusion:**

Inflammation and oxidative stress play a key role in the formation and progression of atherosclerotic plaques. Due to the presence of alkaloids and polyphenols, *B. vulgaris* exhibits strong anti-inflammatory, antioxidant and immunomodulatory effects, making its consumption potentially useful in preventing atherosclerosis.

## Introduction

Cardiovascular diseases (CVDs) are the leading cause of death worldwide, accounting for an estimated 32% of all fatalities that occur each year (Batty et al. 2022; Momeni-Moghaddam et al. 2019). The most prevalent type of CVD is atherosclerosis which is primarily caused by cholesterol buildup and inflammation of the major arteries. Atherosclerosis can eventually result in its clinical consequences, myocardial infarction (MI) and stroke (Björkegren and Lusis 2022). The risk of atherosclerosis has increased due to the prevalence of obesity and diabetes that goes along with it, which is concerning not only in high-income areas but also in developing nations (Soehnlein and Libby 2021). Chronic inflammation and oxidative stress are the key factors in the pathogenesis of atherosclerosis (Higashi 2022). Consumption of foods containing antioxidant and anti-inflammatory properties can be useful for the prevention and treatment of atherosclerosis. *Berberis vulgaris *(*B. vulgaris*), also referred to as barberry, is a well-known herb with numerous medical uses (Kalmarzi et al. 2019). 

The medicinal plant *B. vulgaris* is one of the valuable species of South Khorasan, Iran. The results of studies have shown that the various products of this plant are used individually or in combination with other medicinal species of the region as laxatives, diuretics and strong anti-inflammatories in the treatment of cancer, CVDs and blood pressure diseases. The results of phytochemical investigations have shown that the amount of flavonoid compounds in the plant organs (root, stem, leaf, flower and fruit) is in the range of 8.2-59.9 mg/g equivalent to quercetin, and the amount of total phenol is 16.1-37.8 mg/g equivalent to quercetin. Gallic acid and anthocyanin amounts is very inconstant and in the range of 11.34-153.42 mg/g equivalent of cyanidin-3-glucoside in each organ. Quantitative analysis revealed that berberine alkaloid concentrations in Berberis species varied significantly between plant organs, with concentrations of 1.03% in stem bark and 6.37% in root tissue (Mazandarani et al. 2013).

Several research studies and clinical trials have demonstrated the anti-inflammatory, antioxidant, and immunomodulatory properties of berberine (Shakeri et al. 2024). This study examines the importance of *B. vulgaris* consumption in atherosclerosis improvement.

## Materials and Methods

Databases such as PubMed, Web of Science, and Scopus were considered. The search terms were "*Berberis vulgaris*", "Cardiovascular", "Atherosclerosis", "Inflammation", "Oxidative stress", "Clinic", "Animal", "In vitro", "Cell line" and "Ingredient". The articles were reviewed from December 2004 to December 2024. Congress presentations and unavailable articles were excluded. 

## Results

**Atherosclerosis:** **causes and implications**

CVDs are one of the causes of premature death all over the world, that is why it is called the disease of the century. It is estimated that in 2030, more than 23.3 million people will die annually due to CVDs (Badimon and Vilahur 2014). According to epidemiological studies, about 422.7 million people have been infected with CVDs by 2020 (Luca et al. 2023). Therefore, prevention and treatment of CVDs is extremely important for all worldwide. The increasing number of people living with carotid plaque may represent a significant future burden of CVDs and is therefore a major public health concern worldwide (Song et al. 2020). Since atherosclerosis, the primary pathological process underlying the majority of CVDs, is primarily asymptomatic, it can be challenging to precisely estimate its occurrence. According to epidemiological, clinical, and morphological studies, the atherosclerotic process starts in the womb by thickening the carotid intima-media in fetuses and newborns. This increases the atherosclerotic prevalence and accelerates its progression in children and adolescents. Increased carotid intima thickness is indicative of the start of atherogenesis and provides the framework for the potential build-up of lipids leading to the development of atherosclerotic plaque (Luca et al. 2023).

A large number of environmental and genetic factors play a role in the development of atherosclerosis. Among these factors, age, sex, obesity, smoking, blood pressure and diabetes mellitus can be stated (Momeni-Moghaddam et al. 2022; Stocker and Keaney Jr 2004). As mentioned, several risk factors play a role in the formation and development of atherosclerosis plaques, so this disorder is known as a multifactorial disease (Fava and Montagnana 2018). The first step in the formation of atherosclerosis plaque is the increase in the amount of low-density lipoprotein (LDL) in the bloodstream, which penetrates into the intima of the artery. Continuous exposure to factors such as infections, blood pressure, diabetes, oxidative stress and smoking causes endothelial damage. Endothelial dysfunction allows LDL particles to penetrate further and accumulate in the extracellular matrix (ECM) where they become the target of enzymatic and oxidative changes (Badimon and Vilahur 2014). Among these changes, oxidation by factors such as reactive oxygen species (ROS) can be mentioned. These changes stimulate inflammatory responses (Moore and Tabas 2011). Altered LDLs increase a number of pro-inflammatory responses and affect endothelial cells, causing these cells to express intercellular adhesion molecules (ICAMs) and vascular cell adhesion molecule (VCAM-1), as well as a number of integrins that are involved in the tight adhesion of inflammatory cells to the vessel surface, and in this way, cause the migration of innate immune cells (monocytes, mast cells, neutrophils, natural killer cells (NK) and dendritic cells) to the arterial intima (Badimon and Vilahur 2014; Tziakas et al. 2010). Following the differentiation of macrophages, a large number of receptors are expressed in these cells, which are able to uptake modified and oxidized LDLs, and as a result, macrophages are filled with cholesterol and become foam cells (Badimon and Vilahur 2014). The foam cells produce growth factors and cytokines that stimulate the migration and transfer of vascular smooth muscle cells (VSMC) from the media (the second layer of the artery between the intima and the adventitia) to the intima where these cells divide, produce ECM compounds and thus cooperate in the formation of fibrotic protrusion. A fibrous cap is placed between the lumen of the vessel and the necrotic core. Excessive phagocytosis of apoptotic cells by macrophages can induce endoplasmic reticulum stress, leading to macrophage death. This process triggers the release of lipids and inflammatory mediators, including tissue factor (TF) and matrix metalloproteinases (MMPs). Subsequently, MMPs degrade extracellular matrix (ECM) components, including those within the fibrous cap, thereby increasing plaque vulnerability to rupture. (Badimon and Vilahur 2014). Plaque disintegration causes platelet accumulation and thrombus formation. In addition, the living macrophages present in the plaques play a role in sensitizing the plaque to disintegrate through the secretion of cytokines, proteases and thrombotic/promoting factors (Moore and Tabas 2011). Plaque disintegration progresses slowly with chronic inflammation and lipid-rich plaques are formed in medium and large arteries (Gisterå and Hansson 2017). As mentioned, inflammatory and oxidant factors participate in almost all stages of plaque formation. Therefore, the use of anti-inflammatory and antioxidant agents can be very important for the prevention and treatment of atherosclerosis (Xie et al. 2020). 

Today, various chemical drugs are prescribed for CVDs, but these drugs can cause many side effects for patients in the long run (Vakili et al. 2017; Vakili et al. 2021). Emerging evidence suggests that botanical medicines warrant further investigation as therapeutic alternatives, given their potential benefits and safety considerations. (Mahmoudvand et al. 2017). As demonstrated by epidemiologic research, an abundance of fruits and vegetables lowers the likelihood of acquiring CVDs (Emamat et al. 2020). *B. vulgaris* is a plant that has anti-inflammatory and antioxidant properties. In the following, we review the importance of *B. vulgaris* for the treatment of atherosclerosis.

***Berberis vulgaris*** **: botanical description and nutritional composition**

*Berberis vulgaris* (*B. vulgaris*) is a plant whose medicinal use in Chinese medicine dates back over 200-3000 years ago and belongs to the family of Berberidaceae (Dimitrijević et al. 2020; Rahimi-Madiseh et al. 2017). *B. vulgaris*, also known as "*Zereshk*" in Persian, has long been used as a herbal cure to treat a variety of disorders (Rouhani et al. 2013). The fruit of *B. vulgaris* is also used with rice (Mokhber-Dezfuli et al. 2014). Occasionally, jam and marmalade are made from the fruits (Gundogdu 2013). Jelly, carbonated drinks, honey, candy and chocolate are also made from *B. vulgaris* fruit. Due to the presence of anthocyanin in *B. vulgaris*, it is used instead of artificial dyes. This compound, which is the most important pigment in *B. vulgaris*, has anti-inflammatory properties and is a strong antioxidant due to the presence of hydroxyl groups in its structure (Ardestani et al. 2013). *B. vulgaris* fruits ripen in late summer and autumn, are rich in vitamin C and have a sour taste (Kalmarzi et al. 2019). Beyond its culinary applications, B. vulgaris demonstrates diverse therapeutic properties. Decoction of its leaves has been traditionally employed in treating dysentery, pharyngitis, and vitamin C deficiency. Additionally, extracts from the stem and root bark exhibit antipyretic, antiseptic, choleretic, and diuretic properties.(Mokhber-Dezfuli et al. 2014). Russian sages have also used *B. vulgaris* to treat high blood pressure, inflammation, and abnormal uterine bleeding (Tabeshpour et al. 2017). In addition to the anti-inflammatory and antioxidant effects of *B. vulgaris*, it has other beneficial effects in medicine, such as anti-cancer, liver protection, antimicrobial , anti-parasitic, anti-triglyceridemic, anti-diabetic effects (El-Zahar et al. 2022; Nor 2019).

**Active compounds in** ***B. vulgaris***

**Figure 1:**
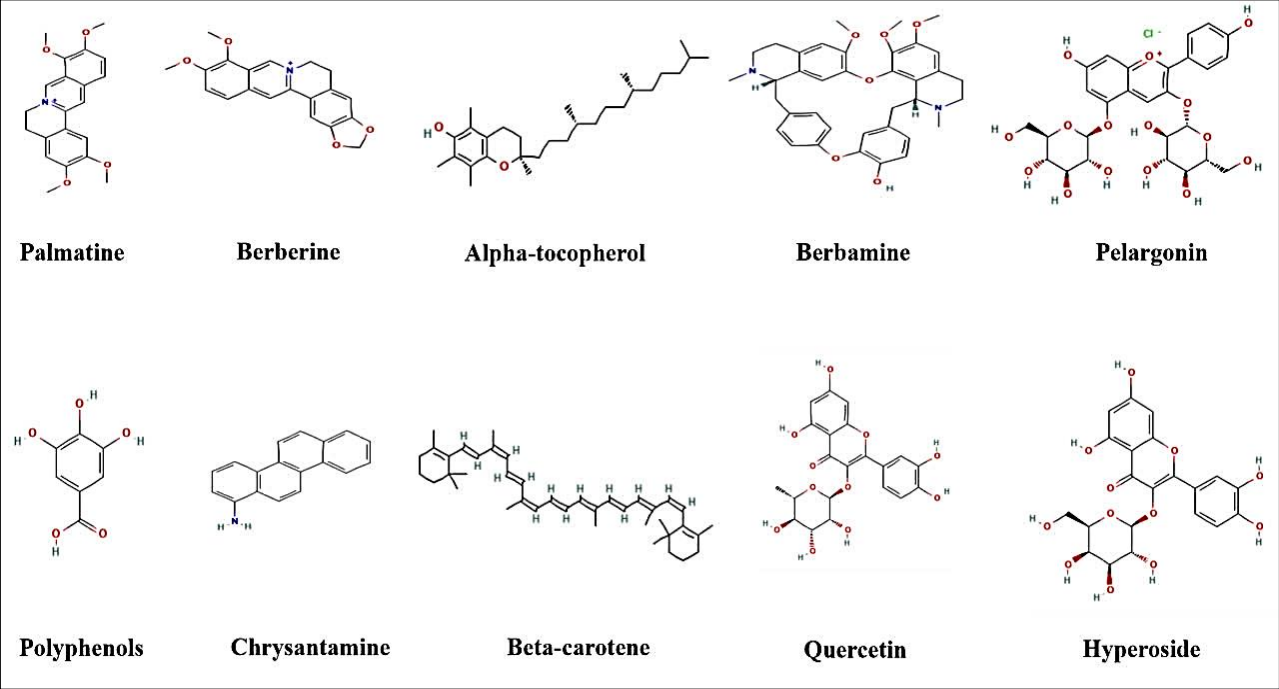
The main B. vulgaris ingredients’ structure

The fruit, bark, leaves, and roots of *B. vulgaris* plant have all long been utilized in Iranian folk medicine (Fatehi et al. 2005). There are currently twenty-two known medicinally significant alkaloids from this plant's fruit, stem leaves, and roots (Arayne et al. 2007). According to research on the plant's chemical makeup, isoquinoline alkaloids including palmatine, berberine, and berbamine are its most significant components (Imanshahidi and Hosseinzadeh 2008). The most significant isoquinoline alkaloid of *B. vulgaris* is berberine; however, because of its limited oral bioavailability (less than 1%), berberine has not been employed as a medicinal agent. Berberine’s weak intestinal penetration and low water solubility are due to its poor oral bioavailability (Mohd Nor et al. 2019; Moustafa et al. 2021). HPLC analyses show that berberine (1.24%) and barbramine (2.5%) are the main alkaloids of the root, bark and stem of *B. vulgaris*. The fruits of *B. vulgaris*, which have a sour taste, contain citric acid, malic acid and tartaric acid. Further, its extract contains beta-carotene, alpha-tocopherol, quercetin, hyperoside, chrysantamine and pelargonin. Also, the seeds, flowers and fruits of *B. vulgaris* have a significant amount of carotenoid and anthocyanin phenolic compounds, vitamin C, vitamin K, oleoresin, pectin, resin, tannin and chelidonic acid (El-Zahar et al. 2022; Kalmarzi et al. 2019). Studies show that phenolic compounds have antioxidant properties. Malic acid and vitamin C have also been shown to have antioxidant qualities and to work in concert with one another (Hanachi and Golkho 2009). Due to the presence of phenolic compounds in *B. vulgaris*, which have antioxidant properties, free radicals can be reduced by using *B. vulgaris*, and as a result, consuming it is useful for resisting oxidative stress (Ardestani et al. 2013; Hemmati et al. 2014; Kalmarzi et al. 2019). Also, it has been found that the highest number of polyphenols is found in leaf, stem and then fruit extracts (Och et al. 2023). Figure 1 shows the main *B. vulgaris* ingredients.

**Evidence of** ***B. vulgaris*** **'s effects on atherosclerosis**

According to Figure 1, *B. vulgaris* has significant antioxidant and anti-inflammatory properties due to the presence of phenols which can prevent the development of atherosclerosis. Considering that inflammation and oxidative stress are among the underlying mechanisms of atherosclerosis, it is important to address this issue. Berberine is one of the primary alkaloids isolated from *B. vulgaris*. In several laboratory models and clinical studies, berberine and *B. vulgaris* have both demonstrated anti-inflammatory, antioxidant, and immunomodulatory properties. 

**Anti-inflammatory and immunomodulatory effects of** ***B. vulgaris***

Studies show that the aqueous extract of *B. vulgaris* has more anti-inflammatory activity than the ethanolic or acetone extract (Moustafa et al. 2021). It has been reported that the primary strategies that prevent inflammation include modulating the immune response of cells to T helper2 (Th2), regulatory T (Treg) cells induction, suppressing inflammatory cytokines (including tumor necrosis factor-alpha (TNF-α), interleukin-1 (IL-1) and Interferon gamma (IFN-γ), and promoting the production of IL-10 and IL-4 (Och et al. 2023). Similarly, it has been indicated that both *B. vulgaris* and berberine can increase the expression of anti-inflammatory cytokines like IL-10 and transforming growth factor β (TGF-β) while reducing inflammatory cytokines like tumor necrosis factor-alpha (TNF-α), interleukin-1β (IL-1β), IL-6, and IL-17. Natural killer cells, lymphocytes, macrophages, and vascular smooth muscle cells secrete pro-atherogenic cytokines such as IL-6, TNF-α, and IL-1. The p38 mitogen-activated protein kinase (p38MAPK)/nuclear factor kappa-light-chain-enhancer of the activated B-cell (NF-κB) pathway is primarily responsible for mediating TNF-α and IL-1 signaling. This pathway impacts nearly all cells involved in atherogenesis by promoting the expression of adhesion molecules, and cytokines, and the migration and mitogenesis of vascular smooth muscle and endothelial cells (Tousoulis et al. 2016). Likewise, Th2 cells secrete cytokines including IL-4 and IL-10 that may modulate inflammation (Moriya 2019). Since two decades ago, clinical data has shown a negative connection between serum TGF-β1 levels and advanced atherosclerosis. Recent experimental research in mice models has also confirmed the atheroprotective role of TGF-β (Singh and Torzewski 2019). Similarly, clinical trials show that *B. vulgaris* reduces the level of pro-inflammatory cytokines IL-6 and TNF-α, which play an important role in the development of atherosclerotic plaques. Moreover, these studies show that the level of C-reactive protein (CRP), as a marker of inflammation, decreases (Vahedi‐Mazdabadi et al. 2023). Emamat et al. showed that *B. vulgaris* consumption not only improved the lipid profile, but also the level of CRP, as an inflammatory marker, decreased in the plasma of these people compared to the control group (Emamat et al. 2020). 

Following the activation and damage of endothelial cells by inflammation pathway, plasma levels of soluble intercellular adhesion molecule-1 (sICAM-1) and monocyte chemoattractant protein 1 (sMCP-1) are increased, which are associated with CVD risk. Consumption of *B. vulgaris* reduces the plasma level of these compounds (Blanco-Colio et al. 2007; Emamat et al. 2021; Palomo et al. 2023). In a study, Karlsen et al. showed that taking *B. vulgaris* supplements in people with CAD, reduces IL-6 and CRP levels, but interestingly, the level of TNF-α increased (Karlsen et al. 2010). Metabolic syndrome is an important risk factor for CVD. In a clinical trial study on people with metabolic syndrome, it was found that *B. vulgaris* significantly reduced LDL and total cholesterol and significantly increased HDL (Zilaee et al. 2014). 

The ethanolic extract of *B. vulgaris* has anti-inflammatory activity *in vitro* and *in vivo* by reducing pro-inflammatory cytokines and factors involved in oxidative stress. Among these factors are TNF-α, IL-6, IL-1β, matrix metalloprotease 9 (MMP9), inducible Nitric oxide synthases (iNOS), monocyte chemoattractant protein 1 (MCP-1) and CRP (Moustafa et al. 2021; Sabariah 2016). MMP-9 levels significantly correlated with the size of the necrotic core of coronary atherosclerotic plaques; thus, MMP-9 is a potent independent predictor of atherosclerotic plaque instability in patients with stable coronary heart disease (CHD). Both plaque instability and a high total carotid artery plaque score were independently correlated with elevated serum MMP-9 levels (Olejarz et al. 2020).  It has been demonstrated that iNOS is essential to the development of atherosclerosis (Liu et al. 2020). Under normal conditions, the inducible isoform of NOS usually disappears from the vasculature. In pathological conditions such as inflammation, sepsis, or oxidative stress, it is induced in blood vessels. An important oxidant that promotes atherosclerosis, i.e. peroxynitrite, is produced more easily when iNOS is induced in the vasculature (Förstermann et al. 2017).

**Anti-oxidant effect of** ***B. vulgaris***

An important risk factor for the beginning and progression of atherosclerosis is oxidative stress which is brought on by excessive production of reactive oxygen species (ROS) (Kattoor et al. 2017; Li et al. 2014a; Momeni-Moghaddam et al. 2020).  Pro-atherogenic mechanisms such as inflammation, impaired lipid metabolism, and endothelial dysfunction are also triggered by ROS. Consequently, an extensive amount of research has gone into determining where oxidative stress originates in blood vessels. Vascular ROS are primarily produced by the enzymes xanthine oxidase, nitric oxide synthases, and mitochondrial electron transport chains (Batty et al. 2022).

Studies show that *B. vulgaris* has antioxidant properties, reduces lipid peroxidation and protects antioxidant enzymes against ROS (Taheri et al. 2012). Studies have demonstrated that the ethanolic extract of *B. vulgaris* exhibits potent antioxidant activity in both *in vitro* and *in vivo* experimental models. (Moustafa et al. 2021). *B. vulgaris* can increase antioxidant enzymes, such as glutathione peroxidase (GPx), catalase (CAT) and superoxide dismutase (SOD) to reduce lipid peroxidation, and DNA damage. Further, *B. vulgaris* can scavenge free radicals for treatment of CVDs (Shakeri et al. 2024; Vahedi‐Mazdabadi et al. 2023). Enzymes are the primary line of defense against free radicals (SOD, CAT, and GPx), while non-enzymes are the secondary line of defense against free radicals (albumin, thiols, β-carotenes, vitamins E and C, etc.) (Radovanovic et al. 2021). SOD, CAT, and GPx are the top three important enzymes in antioxidant defense. Specifically, these enzymes convert superoxide radicals, hydroperoxides, and hydrogen peroxides into innocuous molecules (O_2_, H_2_O_2_, and alcohol) (Ighodaro and Akinloye 2018). 

**Figure 2:**
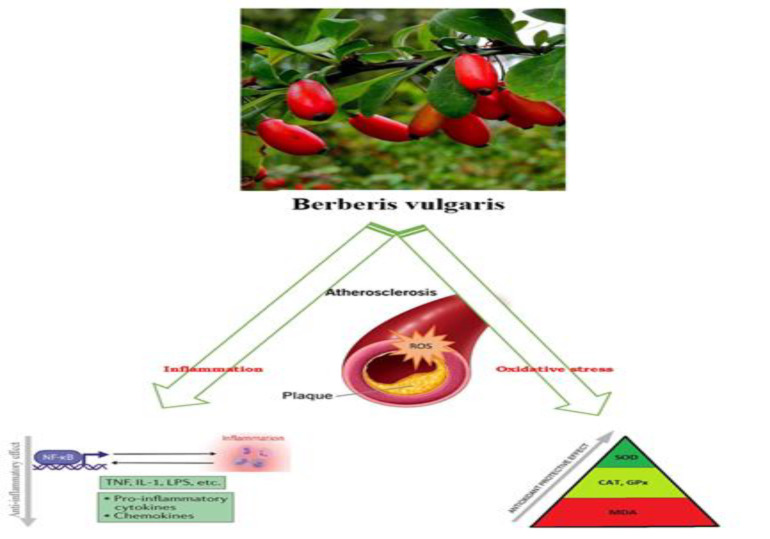
Summary of the B. vulgaris* properties. *Cat= Catalase; GPX= Glutathione peroxidase; Il-1= interleukin-; LPS= Lipopolysaccharide; MDA= Malondialdehyde; NF-κB= Nuclear factor kappa-light-chain-enhancer of activated B cells; SOD= Superoxide dismutase; TNF= tumor necrosis factor

Considering that the occurrence of CVD is high in patients with diabetes, in a study conducted on diabetic rats, it was shown that the consumption of *B. vulgaris* decreased the level of malondialdehyde (MDA), as a marker of lipid peroxidation, and increased the total antioxidant capacity (Hemmati et al. 2015). Figure 2 illustrates the summary of the *B. vulgaris* properties discussed in this review.

## Discussion

*B. vulgaris*, commonly known as barberry, exhibits significant antioxidant properties due to its bioactive compounds, particularly berberine. It enhances the activity of endogenous antioxidant enzymes such as superoxide dismutase (SOD), catalase (CAT), and glutathione peroxidase (GPx), which mitigate oxidative stress (Li et al. 2014b). Additionally, berberine scavenges free radicals and upregulates the nuclear factor erythroid 2-related factor 2 (Nrf2) pathway, increasing the expression of antioxidant enzymes and maintaining cellular redox balance (Hsu et al. 2012). This molecular mechanism highlights its potential in preventing oxidative damage in various diseases (Hsu et al. 2012; Li et al. 2014b).

The anti-inflammatory effects of *B. vulgaris* are primarily mediated through the modulation of cytokine production. Berberine inhibits pro-inflammatory cytokines such as TNF-α, IL-1β, and IL-6 while enhancing the expression of anti-inflammatory cytokines like IL-10 and TGF-β (Kalmarzi et al. 2019). This modulation promotes a Th2 immune response and induces regulatory T cells (Tregs), thereby suppressing inflammatory processes (Zhu et al. 2018). The compound’s ability to inhibit NF-κB activation further contributes to its anti-inflammatory properties (Kalmarzi et al. 2019; Zhu et al. 2018).

*B. vulgaris* also demonstrates immunomodulatory effects by modulating the activity of immune cells. Berberine induces apoptosis in antigen-presenting cells (APCs) and effector cells, reducing their ability to propagate inflammation (Shakeri et al. 2024). It also influences the activity of splenic macrophages and dendritic cells, leading to decreased pro-inflammatory cytokines production and increased anti-inflammatory mediator secretion (Yang et al. 2013). These actions collectively regulate immune responses and maintain immune homeostasis, emphasizing its therapeutic potential in immune-mediated disorders (Shakeri et al. 2024; Yang et al. 2013).

The combined antioxidant, anti-inflammatory, and immunomodulatory effects of *B. vulgaris* underscore its therapeutic promise for managing diseases characterized by oxidative stress and chronic inflammation. By targeting key molecular pathways, berberine offers potential as a natural therapeutic agent for conditions like diabetes, CVDs, and autoimmune disorders. Further research could enhance the understanding and application of this bioactive compound in clinical settings.

*B. vulgaris *and berberine have demonstrated significant effects in mitigating atherosclerosis through various molecular mechanisms. One primary action of berberine is the modulation of lipid metabolism; it upregulates low-density lipoprotein receptors (LDLR) on hepatocyte surfaces, enhancing the clearance of LDL cholesterol from the bloodstream. Additionally, berberine inhibits proprotein convertase subtilisin/kexin type 9 (PCSK9), a protein that degrades LDLR, further promoting LDL cholesterol reduction. This dual action leads to decreased plasma LDL levels, reducing lipid accumulation within arterial walls, a key factor in atherosclerotic plaque formation (Ataei et al. 2022; Lee et al. 2007).

Beyond lipid regulation, berberine exhibits anti-inflammatory properties that contribute to its anti-atherosclerotic effects. It suppresses the expression of pro-inflammatory cytokines and adhesion molecules, reducing monocyte adhesion to endothelial cells, a critical early event in atherogenesis. Furthermore, berberine inhibits the proliferation and migration of VSMCs, processes that contribute to plaque stability and progression. By modulating these cellular activities, berberine not only prevents the initiation of atherosclerotic lesions but also aids in stabilizing existing plaques, reducing the risk of cardiovascular events.

In general, considering that inflammation and oxidative stress play a key role in the formation and progression of atherosclerotic plaques, herbal medicines with anti-inflammatory and antioxidant properties can significantly contribute to its treatment. Due to the presence of alkaloids and polyphenols, *B. vulgaris* exhibits strong anti-inflammatory and antioxidant effects, making its consumption potentially useful in preventing atherosclerosis.
